# A novel [^89^Zr]-anti-PD-1-PET-CT to assess response to PD-1/PD-L1 blockade in lung cancer

**DOI:** 10.3389/fimmu.2023.1272570

**Published:** 2023-09-28

**Authors:** Ander Puyalto, María Rodríguez-Remírez, Inés López, Fabiola Iribarren, Jon Ander Simón, Marga Ecay, María Collantes, Anna Vilalta-Lacarra, Alejandro Francisco-Cruz, Jose Luis Solórzano, Sergio Sandiego, Iván Peñuelas, Alfonso Calvo, Daniel Ajona, Ignacio Gil-Bazo

**Affiliations:** ^1^ Department of Medical Oncology, Clínica Universidad de Navarra, Pamplona, Spain; ^2^ University of Navarra, Cima-University of Navarra, Program in Solid Tumors, Pamplona, Spain; ^3^ Instituto de Investigación Sanitaria de Navarra (IDISNA), Pamplona, Spain; ^4^ Department of Nuclear Medicine, Clínica Universidad de Navarra, Pamplona, Spain; ^5^ Translational Molecular Imaging Unit, Clínica Universidad de Navarra, Pamplona, Spain; ^6^ Department of Pathology, National Institute of Cardiology Ignacio Chavez, Mexico City, Mexico; ^7^ Departamento de Anatomía Patológica y Diagnóstico Molecular, Md Anderson Cancer Center, Madrid, Spain; ^8^ Unidad de Investigación Clínica de Cáncer de Pulmón Hospital Universitario 12 de octubre- Centro Nacional de Investigaciones Oncologicas (H12O-CNIO), Madrid, Spain; ^9^ Department of Oncology, Fundación Instituto Valenciano de Oncología (FIVO), Valencia, Spain; ^10^ Centro de Investigación Biomédica en Red - Cáncer (CIBERONC), Madrid, Spain

**Keywords:** lung adenocarcinoma, inhibitor of differentiation 1, PD-1 inhibition, immuno-PET, pseudoprogression

## Abstract

**Background:**

Harnessing the anti-tumor immune system response by targeting the program cell death protein (PD-1) and program cell death ligand protein (PD-L1) axis has been a major breakthrough in non-small cell lung cancer (NSCLC) therapy. Nonetheless, conventional imaging tools cannot accurately assess response in immunotherapy-treated patients. Using a lung cancer syngeneic mouse model responder to immunotherapy, we aimed to demonstrate that [^89^Zr]-anti-PD-1 immuno-PET is a safe and feasible imaging modality to assess the response to PD-1/PD-L1 blockade in NSCLC.

**Materials and methods:**

A syngeneic mouse model responder to anti-PD-1 therapy was used. Tumor growth and response to PD-1 blockade were monitored by conventional 2-deoxy-2-[^18^F]fluoro-D-glucose ([^18^F]-FDG) PET scans. Additionally, tumor lymphocyte infiltration was analyzed by the use of an [^89^Zr]-labeled anti-PD-1 antibody and measured as ^89^Zr tumor uptake.

**Results:**

Conventional [^18^F]-FDG-PET scans failed to detect the antitumor activity exerted by anti-PD-1 therapy. However, [^89^Zr]-anti-PD-1 uptake was substantially higher in mice that responded to PD-1 blockade. The analysis of tumor-infiltrating immune cell populations and interleukins demonstrated an increased anti-tumor effect elicited by activation of effector immune cells in PD-1-responder mice. Interestingly, a positive correlation between [^89^Zr]-anti-PD-1 uptake and the proportion of tumor-infiltrating lymphocytes (TILs) was found (*Cor* = 0.8; *p* = 0.001).

**Conclusion:**

Our data may support the clinical implementation of immuno-PET as a promising novel imaging tool to predict and assess the response of PD-1/PD-L1 inhibitors in patients with NSCLC.

## Introduction

Lung cancer is the leading cause of cancer deaths (1). Over the last two decades, the development of targeted therapies against certain oncogenic drivers first, and more recently, the use of immune check-point inhibitors (ICIs) have significantly improved the outcomes of metastatic non-small cell lung cancer (NSCLC) patients (2, 3). More specifically, immune modulation through the blockade of the program cell death protein (PD-1)/program cell death ligand protein (PD-L1) axis has obtained the best long-term survival rates ever, with more than 30% of patients being alive at five years (4). ICIs, such as PD-1/PD-L1 monoclonal antibodies (mAbs), reactivate the antigen-specific effector T cells, thus boosting the anti-tumor immune response. Nevertheless, tumor response assessment has become a challenge in NSCLC patients receiving immunotherapy-based systemic regimens.

Although immunohistochemical PD-L1 expression in NSCLC is a predictive biomarker of response to PD-1/PD-L1-inhibiting mAbs, other potential predictive biomarkers such as PD-1 expression in tumor-infiltrating lymphocytes (TILs) or the ability of the PD-1 antibody to reach its target have not been evaluated (5). Moreover, the discovery of new combination therapies are emerging to further improve the efficacy of ICIs (6). Recent reports have demonstrated how targeted therapies can modulate the antigenicity of tumor cells and enhance T cell immune recognition, resulting in a potentially synergistic improvement of the efficacy of this therapeutic approach (7, 8).

Inhibitor of differentiation-1 (*Id1*) is a negative transcription regulator that belongs to the *Id* (*Id1*-*Id4*) gene family (9, 10). In NSCLC patients, Id1 has been associated with poor response and prognosis, as it plays a central role in tumorigenesis, tumor angiogenesis, metastasis, and tumor progression, suppressing the antitumor immune response (11–14). Moreover, Id1 has been described as an immunosuppressor factor involved in the generation of an immunosuppressive tumor microenvironment during tumor progression. In advanced melanoma, Id1 upregulation through tumor growth factor β (TGF-β) has been shown to promote the differentiation of dendritic cells (DCs) to myeloid-derived suppressor cells (MDSCs) and to suppress CD8^+^ T-cell proliferation (12). Furthermore, a recent analysis of Id1 expression from peripheral blood mononuclear cells of stage III and IV melanoma patients, strongly associates high Id1 levels with the presence of phenotypic and immunosuppressive markers in monocytic MDSCs, whereas low Id1 levels are associated with a more immunogenic myeloid phenotype (15). More recently, our group has shown that the combined blockade of *Id1* and PD-1/PD-L1 displays synergistic therapeutic activity in *KRAS*-mutant lung cancer in mouse models. *Id1* downregulation enhanced PD-L1 expression on lung cancer cells surface and increased CD8^+^ T cell infiltration, sensitizing lung tumors that do not respond to PD-1/PD-L1 mAbs (16). Interestingly, we have also demonstrated how a MEK1/2 inhibitor can modulate the immunosuppressive tumor microenvironment of *KRAS*-mutant lung adenocarcinoma (LUAD) tumors though Id1 downregulation (unpublished data).

Advanced imaging methods, specifically computed tomography (CT), positron-emission tomography (PET) and magnetic resonance imaging (MRI), have been established as powerful tools for the staging of lung cancer and the accurate assessment of therapeutic response (17, 18). PET is a well-established 3-dimensional molecular imaging platform that enables non-invasive quantification of the relevant biologic tumor characteristics, using isotope-labelled tracers (19). In NSCLC, the conventional use of 2-deoxy-2-[^18^F]fluoro-D-glucose ([^18^F]-FDG) PET is useful for proper initial staging and response monitoring of patients on systemic treatment. However, this imaging tool may be suboptimal in immunotherapy-treated patients in whom a metabolic uptake increase does not necessarily mean disease progression (20). Response to ICIs is characterized by different patterns, such as progression prior to treatment response (pseudoprogression), hyperprogression, and dissociated responses following treatment. These patterns, however, are not reflected in the Response Evaluation Criteria in Solid Tumors version 1.1 (RECIST 1.1), which is standard for response assessment in oncology. As such, new response evaluation tools are required.

The use of radiotracers other than [^18^F]-FDG is emerging as a non-invasive method to monitor in real time the immune landscape of patients receiving ICIs. This approach is currently under evaluation and may potentially enter routine clinical practice if proven effective (21). Monoclonal antibody-based PET (immuno-PET) is another potential biomarker to 1) verify optimal delivery of targeted agents to tumors and, 2) measure target expression (22, 23).

On these premises, we created an *in vivo* immuno-PET model to profile the immune landscape in a lung cancer mouse model exposed to PD-1/PD-L1 axis blockade. Here we provide key evidence on the preclinical implementation of immuno-PET as a novel imaging tool that may detect antitumor effector immune cells. This strategy could be used to predict and assess the response of PD-1/PD-L1 inhibitors in patients with lung cancer.

## Materials and methods

### Cell lines and reagents

Murine LUAD cell line Lewis lung carcinoma (LLC) was purchased from the American Type Culture Collection (ATCC, Manassas, VA, USA). Cells were cultured in RPMI 1640 medium (Gibco, Waltham, MA, USA) supplemented with 10% fetal bovine serum (FBS) (Thermofisher, Waltham, MA, USA), 1% penicillin-streptomycin (Gibco, Waltham, MA, USA) and HEPES (Lonza, Basel, Switzerland) and routinely tested for *Mycoplasma* using MycoAlert Mycoplasma Detection Kit (Lonza, Basel, Switzerland). Lentiviral production of shRNA against *Id1* (TRCN0000071436; Thermofisher, Waltham, MA, USA) was performed as previously described (13, 16). pLKO.1-scramble (pLKO-Scplasmid #1864, Addgene, Watertown, MA, USA) was used as control.

### Gene expression

Quantification of interleukins: *interleukin-1β* (*Il-1β); tumor necrosis factor alpha (Tnf-α)* and *interferon gamma (Ifn-γ*) gene expression was determined by real-time quantitative PCR as previously described (13, 16). *Gapdh* was used as an endogenous control. The primers designed for RT-PCR are listed in **Table S1**.

### Murine models

All animal procedures were approved by the institutional Committee on Animal Research and Ethics (regional Government of Navarra) under the protocol number CEEA 054-19E1.

This study included 8–12-week-old female C57BL/6J mice (*Id1* ^+/+^) (The Jackson Laboratory, Bar Harbor, ME, USA), and *Id1*-deficient (*Id1^-/-^* or IDKO) mice with C57BL/6J background. *Id1^-/-^* mice were kindly provided by Dr. Robert Benezra (Memorial Sloan-Kettering Cancer Center, New York, NY, USA).

Murine LLC cells (1.5 x 10^6^) with constitutive *Id1* expression (LLC- pLKO-Sc) or *Id1*-silenced (LLC-sh-Id1) cells were injected subcutaneously in the flank of C57BL/6J (*Id1* ^+/+^ and *Id1^-/-^*) mice. Tumor-bearing mice were treated with DPBS or anti-PD-1 monoclonal antibody (RMP1-14, BioXCell, Lebanon, NH, USA) 7, 10 and 14 days after cell inoculation [100 μg per mouse, intraperitoneally (i.p.)]. In the active treatment group, the last injection (day 14) consisted of the anti-PD-1 antibody radiolabeled with zirconium-89 ([^89^Zr]-anti-PD-1). Tumors were measured periodically using a digital caliper (DIN862, Ref 112-G, SESA Tools, Hernani, Spain), and tumor volume was calculated using the formula: volume = π/6 x length x width^2^. At the end of the experiment tumors were harvested and fixed in 4% formaldehyde (pH = 7) for immunohistochemical analyses (Panreac, Castellar del Valles, Spain).

### Antibody conjugation and zirconium-89 radiolabeling

Antibody radiolabeling with zirconium-89 was carried out using a slightly modified version of the protocol described by Vosjan MJ (24). Briefly, a buffer exchange was performed in a small fraction of the monoclonal antibody with a 0.1M bicarbonate buffer (pH = 9), which was then incubated with a 3-fold molar excess of the chelator deferoxamine (DFO) dissolved in 20 µL of dimethyl sulfoxide (Sigma-Aldrich, Saint Louis, MO, USA). After 30 minutes of conjugation, the reaction mixture was purified using a disposable PD-10 desalting column (Healthcare Life Sciences, Eching, Germany). Then 111 MBq of zirconium-89 was added to the anti-PD-1 (RMP1-14, BioXCell, Lebanon, NH, USA) antibody solution (buffered at pH = 7 with HEPES [Lonza, Basel, Switzerland], oxalic acid and sodium bicarbonate) and the reaction was left at room temperature for 30 minutes. Finally, the solution was purified to eliminate any possible non-chelated zirconium-89 by purification with a PD10 column (Healthcare Life Sciences, Eching, Germany).

### *In vivo* PET imaging with [^18^F]-FDG and [^89^Zr]-anti-PD-1

All PET images were acquired on a Mosaic (Philips, Amsterdam, The Netherlands) small animal dedicated tomograph and reconstructed applying dead time, decay, random and scattering corrections into a 128×128 matrix with a 1 mm voxel size. Additionally, on the days that [^89^Zr]-anti-PD1 images were acquired, CT images were performed in a U-SPECT6/E-class (MILabs, Duwboot, The Netherlands) system to obtain the corresponding anatomical correlate of the tumors.

To obtain PET [^18^F]-FDG images, mice were fasted overnight with *ad libitum* access to drinking water. On the day of the study, a dose of 9.3 ± 0.8 MBq was injected intravenously in the tail vein. After 50 minutes, the animals were anesthetized with 2% isoflurane in 100% O_2_ gas and placed prone on the scanner bed for a 15-minute image acquisition. Images with [^89^Zr]-anti-PD-1 were acquired during 30 minutes and 24, 72 and 144 hours post-injection of an intravenous single dose (3.8 ± 0.02 MBq).

PET data were exported and analyzed using the PMOD software (PMOD Technologies Ltd., Adliswil, Switzerland) and transformed to standardized uptake value (SUV) units using the formula SUV = [tissue activity concentration (Bq/cm3)/injected dose (Bq)] × body weight (g). [^18^F]-FDG and ^89^Zr uptake in the tumors was analyzed by drawing volumes of interest (VOI) manually containing the entire tumor, guided by CT when available. Semi-automatic segmentation was then performed including voxels with a value greater than 50% of the maximum value of the tumor. Finally, the average of the SUV values within the semi-automatic VOI was calculated (SUV mean).

### Immunohistochemistry

Immunohistochemistry (IHC) was performed as previously described (13) using an antibody against *Id1* (1:1500, BCH-1/37-2; Biocheck, San Francisco, CA, USA). IHC was performed to study the expression of CD3 (1:150, ab16669; Abcam, Cambridge, United Kingdom), CD8 (1:400, #98941S; Cell Signaling, Danvers, MA, USA) and CD4 (1:400, #25229S; Cell Signaling, Danvers, MA, USA), as previously described (16). Slides were scanned using the Aperio Digital Scanner (Leica, Wetzlar, Germany) and analyzed with Image J software (NIH, Bethesda, MD, USA).

### Multiplex-VECTRA

For multispectral immunophenotyping in mouse tumors, the murine-specific NEL810001KT Opal kit (Akoya, Marlborough, MA, USA) was used following the manufacturer’s instructions and as previously described (16), with additional markers. This kit includes the Alexa Fluor tyramides Opals 520, 570 and 690 and spectral DAPI. Opals 650 (R55503) was not included in the kit, so was purchased separately from Akoya. The following primary antibodies were used: anti-CD3 (1:150, ab16669; Abcam, Cambridge, United Kingdom), anti-CD4 (1:200, #25229S; Cell Signaling, Danvers, MA, USA), anti-CD8 (1:400, #98941S; Cell Signaling, Danvers, MA, USA) and anti-Id1 (1:1000, BCH-1/37-2; Biocheck, San Francisco, CA, USA). Vectra Polaris Automated Quantitative Pathology Imaging System and the Phenochart and InForm 2.4 software (Akoya, Marloborough, MA, USA) were used for sample scanning, spectral unmixing, and quantification of signals. Data were given as number of cells with a specific immunophenotype/total number of cells.

### Statistical analysis

A Shapiro–Wilk test was conducted to analyze the normality of the samples. Statistical significance was assessed using a Mann–Whitney *U* test (for comparisons between two groups) and one-way ANOVA followed by a *post hoc* test, or Kruskal-Wallis followed by a *post hoc* test (for comparisons between different groups). The relationship between zirconium-89 uptake and immune cell infiltration was analyzed using Pearson correlations. A *p*-value of <0.05 was considered statistically significant. Statistical analyses were performed using Prism software version 8.0 (GraphPad, San Diego, CA, USA).

## Results

### [^18^F]-FDG-PET scan fails to identify PD-1/PD-L1 blockade antitumor response

In order to explore the limitations of conventional [^18^F]-FDG-PET scans for accurately assessing the response to immunotherapy treatments, we used a lung cancer syngeneic mouse model exposed to PD-1/PD-L1 axis blockade, as previously published (16).

**Figure 1:**
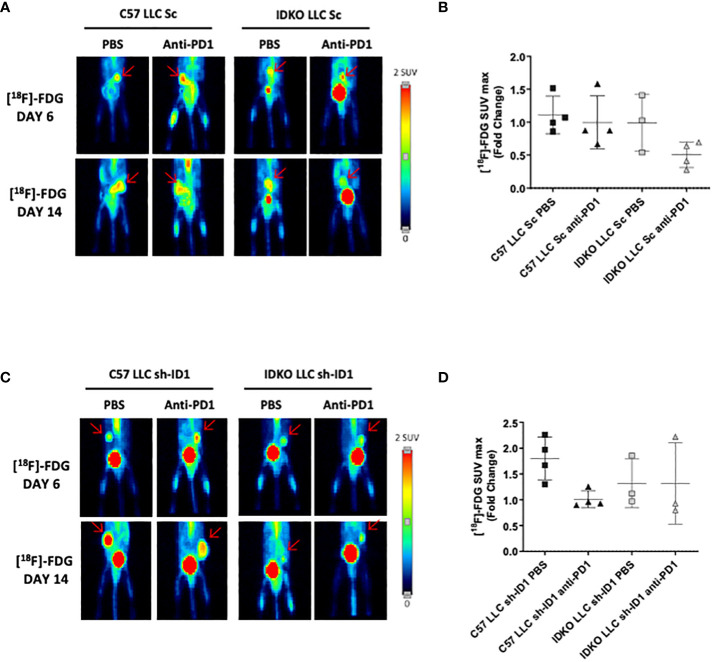
[^18^F]-FDG does not detect the antitumor effect of PD-1 blockade in LLC tumors. **(A)** Representative PET images at days 6 and 13 of ^1^[^18^F]-FDG uptake in LLC cells (LLC Sc) injected in *Id1^+/+^* (C57BL/6J) or *Id1^-/-^* (IDKO) (n = 4) mice groups. **(B)** Quantification of [^18^F]-FDG SUVmax representing the fold change between the uptake at days 6 and 13 of the four mice groups described in **(A)**. **(C)** Representative PET images of [^18^F]-FDG uptake at days 6 and 13 of *Id1* silenced LLC cells (LLC sh-ID1) injected in *Id1^+/+^* (C57BL/6J) or *Id1^-/-^* (IDKO) (n = 4) mice groups. **(D)** Quantification of [^18^F]-FDG SUVmax representing the fold change between the uptake at days 6 and 13 of the four mice groups described in **(C)**. Error bars denote SD.

We investigated the synergistic impact of anti-PD-1 treatment combined with *Id1* abrogation at the host tumor microenvironment. This therapeutic combination reduced LLC tumor growth. However, no statistically significant differences were observed between mice with constitutive expression of *Id1* (C57) and mice with *Id1* silenced at the host microenvironment (IDKO) (C57-LLC_Sc/PBS: 1709 [1171-2025]; C57-LLC_Sc/anti-PD-1: 1079 [489.5-1911]; IDKO-LLC_Sc/PBS: 874.2 [275.7-1473]; IDKO-LLC_Sc/anti-PD-1: 246.2) (**Supplementary Figures S1A, B**). In contrast with tumor volume, [^18^F]-FDG uptake did not show any significant tumor metabolic response (C57-LLC_Sc/PBS: 1.111 [0.894-1.405]; C57-LLC_Sc/anti-PD-1: 0.997 [0.715-1.404]; IDKO-LLC_Sc/PBS: 0.992 [0.541-1.409]; IDKO-LLC_Sc/anti-PD-1: 0.505 [0.311-0.680]) (**Figures 1A, B**).

We also studied the impact of anti-PD-1 blockade after complete inhibition of *Id1* expression (tumor cells and host microenvironment). *Id1* silencing (alone or in combination with anti-PD-1 therapy) significantly reduced tumor growth (C57-LLC_shID1/PBS: 947.6 [540.3-1334]; C57-LLC_shID1/PD-1: 1255 [1130-1359]; IDKO-LLC_shID1/PBS: 312.9 [30.07-596.7]; IDKO-LLC_shID1/PD-1: 47.09 [10.96-100.3], and *p*-values for the groups were as follows: C57-PBS/IDKO-PBS *p* = 0.0352; C57-PBS/IDKO-PD-1 *p* = 0.0066; C57-PD-1/IDKO-PBS *p* = 0.0027; C57-PD-1/IDKO-PD-1 *p* = 0.0007) (**Supplementary Figures S1C, D**). Of note, no significant differences [^18^F]-FDG uptake were observed among the different mice groups (C57-LLC_shID1/PBS: 1.8 [1.393-2.187]; C57-LLC_shID1/anti-PD-1: 1 [0.904-1.179]; IDKO-LLC_shID1/PBS: 1.3 [0.972-1.857]; IDKO-LLC_shID1/anti-PD-1: 1.3 [0.799-2.222]) (**Figures 1C, D**).

Taken together, these results suggest that the [^18^F]-FDG-PET scan fails to assess antitumor immune response upon PD-1/PD-L1 blockade in this animal model.

### [^89^Zr]-anti-PD-1 uptake correlates with tumor-infiltrating CD8^+^ T cells

Given the limitations observed with conventional [^18^F]-FDG-PET to monitor PD-1/PD-L1 blockade antitumor response, we used an additional novel radiotracer labeling protocol based on the use of anti-PD-1 mAb with ^89^Zr. PD-L1 is widely expressed in both immune cells, as well as in tumor cells, whereas PD-1 is mainly expressed on the surface of activated T cells, B cells, and monocytes (25). Therefore, we hypothesized that [^89^Zr]-anti-PD-1 signal may predict and correlate with tumor infiltrating lymphocytes.

**Figure 2:**
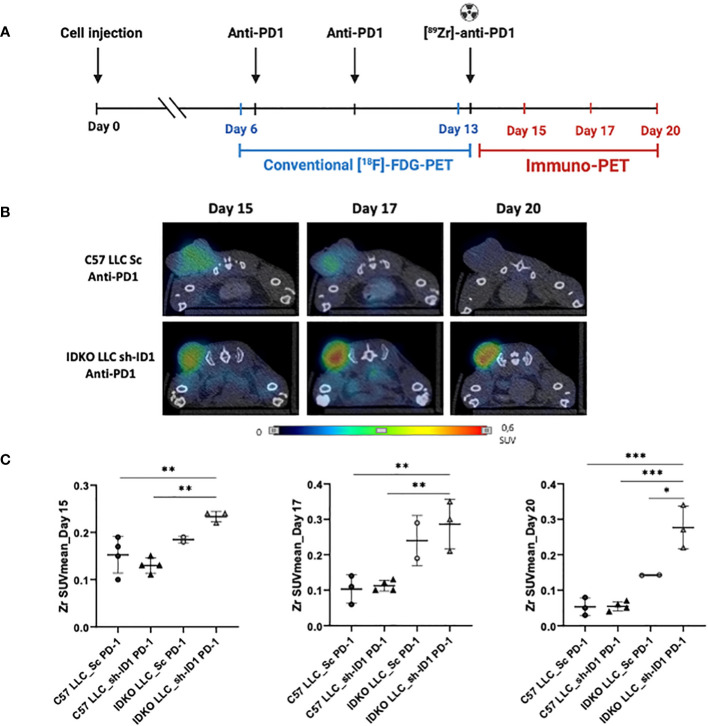
[^89^Zr]-anti-PD-1 allows an accurate evaluation of tumor response to immunotherapy in a lung cancer mouse model. **(A)** Outline of the mouse model. LLC [sh_Control (Sc) and *Id1* silenced cells (sh-ID1)] were subcutaneously injected in *Id1 ^+/+^* C57BL/6J (C57) and *Id1 ^-/-^* C57BL/6J (IDKO) mice, and animals were treated with PBS or an anti-PD-1 mAb (days 7, 10; RPM-14 100mg, intraperitoneally) and with [^89^Zr]-anti-PD-1 (day 14; 100mg, intraperitoneally) (n=4 mice per group). Tumor volume was measured using a caliper (mm^3^), using [^18^F]-FDG-PET scan analyses (days 6 and 13) or using [^89^Zr]-anti-PD-1 PET scans analysis (days 15, 17 and 20 after LLC inoculation). **(B)** Representative PET-CT images of ^89^Zr signal at days 15, 17 and 20 after LLC inoculation. **(C)** ^89^Zr signal LLC Sc and sh-ID1 cells injected in C57 and IDKO treated with anti-PD-1 at days 15, 17, and 20 after LLC inoculation. Asterisks denote significance (*p < 0.05, **p < 0.005, ***p < 0.001), and error bars denote SD.

Anti-PD-1 treated mice, in their last treatment dose, received an anti-PD-1 mAb labelled with ^89^Zr, and the ^89^Zr signal was sequentially measured by immuno-PET (**Figure 2A**). ^89^Zr uptake was significantly higher in mouse tumors when *Id1* was inhibited at both the host microenvironment and tumor cells. Interestingly, this uptake was sustained until the end point of the experiment; (*p*-values for the groups were as follows: Day 15, C57 LLC_Sc *p* = 0.0133; C57 LLC_sh-ID1 *p* = 0.0032; Day 17, C57 LLC_Sc *p* = 0.0075; C57 LLC_sh-ID1 *p* = 0.0069; Day 20, C57 LLC_Sc *p* = 0.0002; C57 LLC_sh-ID1 *p* = 0.0001; IDKO LLC_Sc *p* = 0.0102) (**Figures 2B, C**; **Table 1**).

**Table 1:** [^89^Zr]-anti-PD-1 uptake from all mice treated with [^89^Zr]-anti-PD-1 at days 15, 17 and 20 after cell inoculation.

[^89^Zr]-anti-PD-1 uptake (Day)	C57 LLC_Sc		C57 LLC_sh-ID1		IDKO LLC_Sc		IDKO LLC_sh-ID1	
	Mean	Interquartile range	Mean	Interquartile range	Mean	Interquartile range	Mean	Interquartile range
***15***	0.1467	0.1-0.19	0.13	0.115-0.145	0.185	0.18-0.19	0.2333	0.22-0.24
***17***	0.1033	0.06-0.14	0.1125	0.1-0.1275	0.24	0.19-0.29	0.2867	0.21-0.35
***20***	0.0533	0.03-0.08	0.055	0.0425-0.0675	0.1524	0.142-0.143	0.2767	0.22-0.34

**Figure 3:**
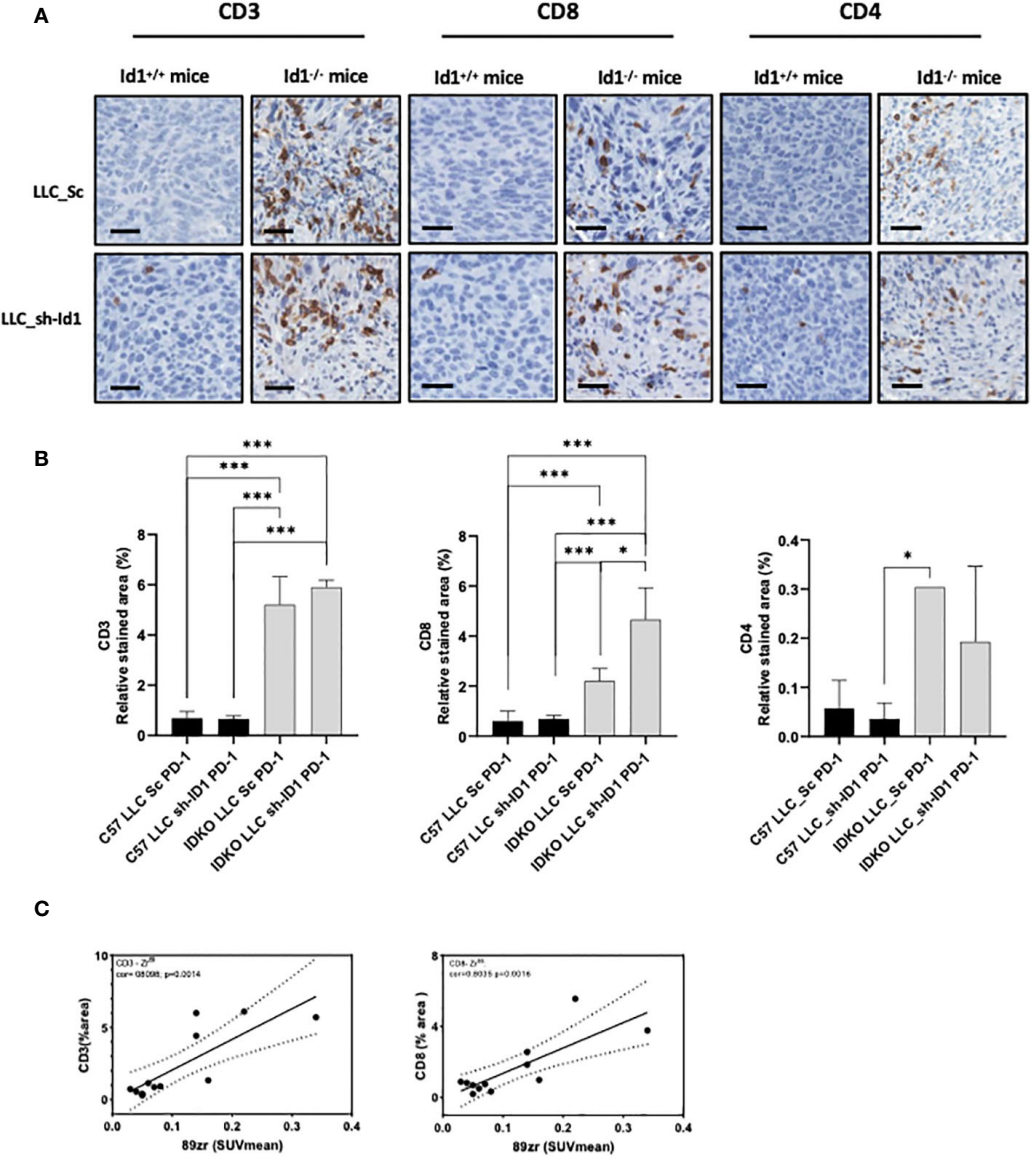
[^89^Zr]-anti-PD-1 uptake correlates with immune T cell infiltration. **(A)** Representative IHC images illustrating: Left: CD3^+^ T cells; Middle: CD8^+^ T cells; Right: CD4^+^ T cells; Scale bar: 200μm. **(B)** Quantification of the relative stained area of: Left: CD3^+^ T cells; Middle: CD8^+^ T cells; Right: CD4^+^ T cells. **(C)** Correlation between ^89^Zr uptake at day 20 after LLC inoculation and: Left: CD3^+^ T cells proportion area (*Cor* = 0.8); Right: CD8^+^ T cells proportion area (*Cor* = 0.8). Asterisks denote significance (*p < 0.05, ***p < 0.001), and error bars denote SD.

The immune populations in tumor samples were then characterized. *Id1* blockade in the tumor microenvironment (*Id1* ^-/-^ mice) significantly enhanced CD3^+^ T cell infiltration in tumor samples as compared to *Id1* ^+/+^ mice. However, no significant differences were observed when additional *Id1* genetic silencing in LLC cells was added to *Id1* inhibition in the tumor microenvironment in terms of CD3^+^ T cells infiltration in tumor samples; (*p* > 0.0001) (**Figures 3A, B**; **Supplementary Figure S2A**; **Table 2**). Similarly, the effector CD8^+^ T cells tumor infiltration was significantly increased among *Id1*-deficient animals as compared to *Id1*-expressing mice. In contrast with the observations made regarding CD3^+^ T cell infiltration, CD8^+^ T cell infiltration was significantly higher in mice with complete *Id1* depletion (tumor cells plus tumor microenvironment) (*p* > 0.0001; IDKO LLC_Sc/sh-ID1 *p* = 0.0497) (**Figures 3A, B**; **Supplementary Figure S2B**; **Table 2**). *Id1* abrogation also enhanced infiltration of CD4^+^ T cells (*p* = 0.0488) (**Figures 3A, B**; **Supplementary Figure S2C**; **Table 2**). Interestingly, a correlation was found between [^89^Zr]-anti-PD-1 uptake signal detected with immuno-PET and the presence of CD3^+^ (*Cor* = 0.8098; *p* = 0.0014) and CD8^+^ T cells (*Cor* = 0.8035; *p* = 0.0016) analyzed in tumor samples by IHC (**Figure 3C**).

**Table 2:** Immunohistochemical analysis of markers of tumor-infiltrating lymphocytes from all mice treated with [^89^Zr]-anti-PD-1.

IHC marker	C57 LLC_Sc [^89^Zr]-anti-PD-1		C57 LLC_sh-ID1 [^89^Zr]-anti-PD-1		IDKO LLC_Sc [^89^Zr]-anti-PD-1		IDKO LLC_sh-ID1 [^89^Zr]-anti-PD-1	
	Median	Interquartile range	Median	Interquartile range	Median	Interquartile range	Median	Interquartile range
***CD3***	0.8551	0.228-1.482	0.659	0.463-0.855	5.209	-4.831-15.25	5.906	3.395-8.418
***CD8***	0.603	-0.024-1.229	0.692	0.478-0.906	2.202	-2.298-6.703	4.666	-6.681-16.01
***CD4***	0.057	-0.033-0.148	0.036	-0.016-0.087	0.304		0.193	-1.186-1.572

Collectively, the Zr uptake and its correlation with TILs in anti-PD-1 responder mice, suggest that [^89^Zr]-anti-PD-1 immuno-PET allows an accurate evaluation of tumor response to immunotherapy.

### *Id1* abrogation enhanced proinflammatory interleukins expression

**Figure 4:**
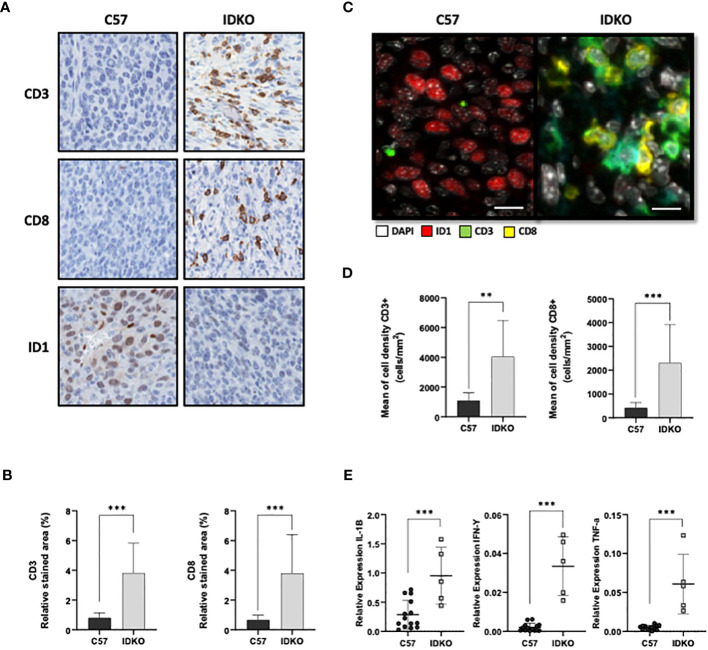
*Id1* inhibition at the tumor-microenvironment promotes proinflammatory interleukin expression and T cell infiltration. **(A)** Representative IHC images illustrating CD3^+^ T cells, CD8^+^ T cells and Id1^+^ cells of LLC cells inoculated in C57 and IDKO mice. Scale bar: 200μm. **(B)** Left: Quantification of proportion of relative stained area of CD3^+^ T cells. Right: Quantification of the proportion of relative stained area of CD8^+^ T cells of tumor samples illustrated in **(A)**. **(C)** Representative images of multiplex immunofluorescence staining panel with nuclei (white), Id1 (red), CD3 (green), CD8 (yellow) of LLC cells inoculated in C57 and IDKO mice. Scale bar: 200μm. **(D)** Left: Quantification of multiplex immunofluorescence staining of CD3^+^ T cells. Right: Quantification of multiplex immunofluorescence staining of CD8^+^ T cells of tumor samples illustrated in **(C)**. **(E)** Relative mRNA expression levels of *Il-1b*, *Ifn-γ* and *Tnf-α* in LLC tumors in C57 and IDKO mice. Asterisks denote significance (*p < 0.05, ***p < 0.001), and error bars denote SD.

In order to explore the clinical relevance of *Id1* depletion at the tumor microenvironment, we analyzed immune populations in tumors from mice responding to anti-PD-1 therapy (in *Id1*-silenced host microenvironment) and non-responding mice (mice with constitutive *Id1* expression). *Id1* absence in the tumor microenvironment in *Id1*-deficient mice significantly increased the tumor infiltration of CD3^+^ T cells, and more importantly, effector CD8^+^ T cells, as compared to *Id1*-expressing mice (CD3^+^ T cells *p* > 0.0001; CD8^+^ T cells *p* > 0.0001) (**Figures 4A, B**; **Table 3**). Additionally, quantitative multiplexed IHC (labelling CD3, CD8 and Id1) revealed an increase in the proportion of CD3^+^ TILs and CD8^+^ T lymphocytes in tumors derived from *Id1* knockout mice (CD3^+^ T cells *p* = 0.0011; CD8^+^ T cells *p* > 0.0001) (**Figures 4C, D**; **Table 3**).

**Table 3:** Immunohistochemical and multispectral immunophenotyping analysis of markers of tumor-infiltrating lymphocytes from anti-PD-1 non-responding and responding mice.

IHC marker	IHC				Multiplex-IHC			
	C57		IDKO		C57		IDKO	
	Mean	Interquartile range	Mean	Interquartile range	Mean	Interquartile range	Mean	Interquartile range
**CD3**	0.793	0.55-0.97	3.817	1.63-5.854	1075	542.3-1636	4053	1973-6500
**CD8**	0.656	0.385-0.866	3.803	1.934-5.112	441.2	246.3-549.7	2228	1162-3935

Immunohistochemical (IHC); multispectral immunophenotyping (Multiplex-IHC); anti-PD-1 non-responding mice (C57); anti-PD-1 responding mice (IDKO).

We also explored the expression of the cytokines *Il-1β, Tnf-α* and *Ifn-γ* using RT-PCR. *Id1* absence in the host microenvironment enhanced the expression of *Il-1b*, *Ifn-γ* and *Tnf-α*, factors implicated in immune T cell activation (*Il-1b*, C57: 0.28 [0.078-0.54]; IDKO: 0.95 [0.5-1.45], *p* > 0.0001; *Ifn-γ*; C57: 0.002 [0.0004-0.003]; IDKO: 0.033 [0.017-0.047], *p* > 0.0001; *Tnf-α*, C57: 0.005 [0.002-0.007]; IDKO: 0.06 [0.029-0.093], *p* = 0.0002) (**Figure 4E**).

Collectively, these data show that *Id1* absence in the host microenvironment can induce tumor TIL infiltration. More importantly, we also demonstrate that *Id1* inhibition may enhance proinflammatory interleukin expression implicated in immune T cell activation.

## Discussion

Tumor response assessment has become a challenge in NSCLC patients receiving immunotherapy-based systemic regimens. According to their particular mechanisms of action based on T-cell activation, response to ICIs is characterized by different patterns, such as progression prior to treatment response (pseudoprogression), hyperprogression, and dissociated responses following treatment. These patterns, however, are not reflected in the Response Evaluation Criteria in Solid Tumors version 1.1 (RECIST 1.1), which is the standard for response assessment in oncology. Therefore, new response evaluation tools are required.

In this paper, we propose the use of a PET-CT scan based on an [^89^Zr]-anti-PD-1 radiotracer as a strategy to overcome the limitations of conventional [^18^F]-FDG-PET scans in assessing immunotherapy antitumor responses. Our results show that an [^89^Zr]-anti-PD-1-PET-CT could accurately assess tumor response to ICIs and may constitute a potential biomarker which directly labels effector T cells and predicts the efficacy of PD-1 inhibition in real time, non-invasively and safely.

[^18^F]-FDG-PET-CT is a powerful tool for monitoring lung cancer initial staging and antitumor response due to its ability to detect small metastatic lesions and regional lymph node tumor spread more accurately than the conventional CT and MRI imaging methods (26, 27). A number of clinical studies have shown that alterations in metabolic activity, expressed as changes in SUV during induction therapy or at interim evaluation, are associated with tumor response. In NSCLC, a reduction of SUVmax below 2.5 after 2-4 conventional chemotherapy cycles has been considered a predictor of future response associated with a substantially higher median time to recurrence (28). Similarly, when the treatment studied is a targeted therapy, such as the EGFR tyrosine-kinase inhibitor erlotinib, a reduction in SUVmax measured by [^18^F]-FDG-PET is also clearly associated with durable therapeutic responses in NSCLC patients (29). In contrast with conventional chemotherapy or targeted therapies, the response pattern of patients treated with ICIs may be substantially different, with some patients developing hyperprogression or pseudoprogression (30). [^18^F]-FDG is a reliable radiotracer for monitoring glucose metabolism. However, changes in glucose metabolism are not restricted to tumor cells, and anti-tumor immune-related cells can similarly show major changes in the glucose metabolism when they are externally activated. ICIs reactivate effector T cells, boosting immune and natural inflammatory response and conventional [^18^F]-FDG-PET scans have shown inaccuracies when examining responses to these drugs (31). We also observed limitations with [^18^F]-FDG-PET in our lung cancer murine model when assessing tumor response to PD-1/PD-L1 axis blockade among mice responding to anti-PD-1 blockade and *Id1* inhibition. In previous studies, we have shown that *Id1* complete abrogation at both host microenvironment and tumor cells sensitized lung tumors to anti-PD-1 therapy, substantially reducing tumor growth (16). However, no significant differences were observed at the SUVmax ^1^[^18^F]-FDG uptake level between responders and non-responders to immunotherapy.

According to RECIST, the tumor burden may transiently increase and then decrease as treatment continues, due to an immune reaction between tumor cells and host immune cells (32). The immune-related Response Evaluation Criteria in Solid Tumor (irRECIST) has been proposed as an update to the RECIST criteria for the assessment of response to ICIs (33). For PET response evaluation, different response criteria have also been proposed, such as the EORTC (European Organisation for Research and Treatment of Cancer) and PERCIST (Positron Emission Tomography Response Criteria in Solid Tumors) (34, 35). Nevertheless, the specific mechanism of action of ICIs has created unique [^18^F]-FDG-PET-CT response patterns, which make it difficult to determine these responses using the PERCIST criteria. This has led to the elaboration of new multiple criteria specifically addressing ICIs response, such as PECRIT (Early Prediction of Response to Immune Checkpoint Inhibitor Therapy) or PERCIMT (Response Evaluation Criteria for Immunotherapy) (36). However, the lack of harmonization hampers the wider adoption of molecular imaging as a more accurate and reliable tool for response assessment to immune-modulating agents in cancer patients.

Immuno-PET is a whole-body, non-invasive molecular imaging technique that combines the high resolution, quantitative ability and sensitivity of PET with the specific binding property of mAbs (37). Immuno-PET has become a relevant novel imaging tool in the molecular evaluation of hematological malignancies and solid tumors (22, 37). Two approaches have been made to date using radiolabeled immunotherapy mAbs (pembrolizumab, nivolumab) and probes directed to target immune biomarkers (PD-1/PD-L1, CD8) (38–40). In NSCLC, [^89^Zr]-nivolumab and [^18^F]-BMS-986192 were studied in 13 patients. The median [^18^F]-BMS-986192 and [^89^Zr]-nivolumab SUVpeak was higher for lesions with ≥50% tumor PD-L1 than for lesions with <50% expression (8.2 vs. 2.9, *p* = 0.018); and aggregates of PD-1 positive tumor-infiltrating immune cells (7.0 vs. 2.7, *p* = 0.03) (38).. More recently, another study was performed in 12 NSCLC patients using [^89^Zr]-pembrolizumab immuno-PET before pembrolizumab monotherapy. This study showed that [^89^Zr]-pembrolizumab PET-CT is a safe and feasible imaging modality. Moreover, [^89^Zr]-pembrolizumab uptake was higher among responding patients than non-responding patients, although no significant differences were observed (SUVpeak 11.4 vs 5.7, *p* = 0.066) (40). The NCT02760225 clinical trial, has recently evaluated the use of [^89^Zr]-pembrolizumab to assess tumor uptake, antibody biodistribution and patient outcome in patients with locally advanced or metastatic melanoma and NSCLC. Interestingly, they found that [^89^Zr]-pembrolizumab uptake not only correlated with response to anti-PD-1 therapy (*p* = 0.014), but also with progression-free survival (PFS) (*p* = 0.0025) and overall survival (OS) (*p* = 0.026) (23). More recently, a study performed in patients with head and neck squamous cell carcinoma and NSCLC showed [^89^Zr]-BI 754111, a LAG-3 targeting mAb, to be a predictive imaging biomarker for anti-LAG-3 therapy (21).

Similarly, in our lung cancer mouse model we show that [^89^Zr]-anti-PD-1 uptake in tumor lesions was significantly higher among treatment-responding *Id1* knock-out mice, than among non-responding mice maintaining a constitutive *Id1* expression. More importantly, the novelty of our study relies on the demonstration of a clear correlation between that [^89^Zr]-anti-PD-1 uptake and the proportion of TILs (CD3^+^ T and effector CD8^+^ T cells) infiltrating tumor lesions and with a proinflammatory tumor microenvironment. As we previously demonstrated, *Id1* and PD-1 combine blockade synergy was generated mainly through CD8^+^ T cells infiltration (16). We also showed in our mouse model an increase in the presence of CD8^+^ T cells and the correlation with the [^89^Zr]-anti-PD-1 uptake in anti-PD-1 responder mice. Consistently, we found a significant upregulation of proinflammatory cytokines in responding mice, such as *Ifn-γ* and *Tnf-α*, that are involved in CD8^+^ T cell tumor killing activity (41, 42). Moreover, the expression of *Ifn-γ* and *Il-1β* in tumor samples of anti-PD-1 responder mice, might suggest the infiltration by other antitumor immune system cells (43, 44).Therefore, taken together these data we provide a potential mechanistic explanation for the enhanced tumor response observed.

The main limitation of our study is the relatively small tumor sample size analyzed, since most mice with combined complete *Id1* genetic abrogation (*Id1*-deficient mice injected with *Id1*-silenced tumor cells) and PD-1 blockade showed complete regression of their syngeneic lung cancer tumors, so they could not be measured. Moreover, our experiments were only performed in immunologically competent murine models, so these results should be confirmed in humanized murine models.

We have demonstrated the utility of [^89^Zr]-anti-PD-1 immuno-PET as a novel imaging tool for the non-invasive, real-time detection of antitumor effector TILs in a lung cancer mouse model responder to anti-PD-1 therapy. In this regard, [^89^Zr]-anti-PD-1 (immuno-PET) may probably perform similarly well in other lung cancer subtypes for which immunotherapy has been approved in frontline treatment or as subsequent lines (*KRAS* or *BRAF* mutant lung tumors).

## Conclusions

This study proposes the potential use of [^89^Zr]-anti-PD-1 immuno-PET as a safe and feasible imaging modality to monitor PD-1/PD-L1 inhibitor antitumor response in NSCLC. We show that [^89^Zr]-anti-PD-1 uptake is significantly higher in anti-PD-1-responding mice as compared to non-responding mice. Moreover, our data may confirm the capacity of [^89^Zr]-anti-PD-1 immuno-PET to label PD–expressing immune cells, such as effector CD8^+^ TILs. Nevertheless, further investigation is warranted.

## Data availability statement

The raw data supporting the conclusions of this article will be made available by the authors, without undue reservation.

## Ethics statement

The animal study was approved by Committee on Animal Research and Ethics (regional Government of Navarra). The study was conducted in accordance with the local legislation and institutional requirements.

## Author contributions

AP: Formal Analysis, Investigation, Methodology, Validation, Visualization, Writing – original draft, Writing – review & editing. MR-R: Formal Analysis, Investigation, Validation, Visualization, Writing – original draft, Writing – review & editing. IL: Data curation, Validation, Visualization, Writing – review & editing. FI: Formal Analysis, Writing – review & editing. JS: Writing – review & editing. ME: Data curation, Writing – review & editing. MC: Data curation, Formal Analysis, Writing – review & editing. AV-L: Formal Analysis, Writing – review & editing. AF-C: Data curation, Formal Analysis, Writing – review & editing. JS: Data curation, Formal Analysis, Writing – review & editing. SS: Writing – review & editing, Project administration, Supervision. IP: Project administration, Supervision, Writing – review & editing. AC: Project administration, Supervision, Writing – review & editing. DA: Project administration, Supervision, Writing – review & editing. IG-B: Conceptualization, Funding acquisition, Project administration, Supervision, Writing – original draft, Writing – review & editing.
