# Supplementary material for: A novel [89Zr]-anti-PD-1-PET-CT to assess response to PD-1/PD-L1 blockade in lung cancer
Source: Front Immunol. 2023 Sep 28;14:1272570. doi: 10.3389/fimmu.2023.1272570 (PMC10569300; doi:10.3389/fimmu.2023.1272570)
Supplement: Supplementary file 1 [file DataSheet_1.docx]

Supplementary Material

## Supplementary Figure 1

**Supplementary Figure 1.** **Effect of combined Id1 and PD-1 blockade in both tumor microenvironment (IDKO mice) and tumor cells in a LUAD syngeneic model.** A) Tumor growth of LLC cells (LLC Sc) injected in *Id1^+/+^* (C57BL/6J) or *Id1^-/-^* (IDKO) mice (n = 4). B) Tumor volumes at day 16. C) Tumor growth of *Id1* silenced LLC cells (LLC sh-ID1) injected in *Id1^+/+^* (C57BL/6J) or *Id1^-/-^* (IDKO) mice (n = 4). D) Tumor volumes at day 16. Asterisks denote significance (**p* < 0.05, ***p* < 0.005, ****p* < 0.001, *****p* < 0.0001), and error bars denote SD.

## Supplementary Figure 2

**Supplementary Figure 2. Id1 and PD-1 combine blockade enhanced T cell infiltration. A-C)** Representative IHC images illustrating: CD3^+^ T cells (A); CD8^+^ T cells (B); Right: CD4^+^ T cells (C). Scale bar: 10μm.
