# Supplementary material for: A novel [89Zr]-anti-PD-1-PET-CT to assess response to PD-1/PD-L1 blockade in lung cancer
Source: Front Immunol. 2023 Sep 28;14:1272570. doi: 10.3389/fimmu.2023.1272570 (PMC10569300; doi:10.3389/fimmu.2023.1272570)
Supplement: Supplementary file 2 [file Table_1.docx]

Table S1. Real Time-PCR Primers

|  | **Fw (5’→3’)** | **Rv (5’→3’)** |
| --- | --- | --- |
| Murine *Il-1a* | TTGGTTAAATGACCTGCAACA | GAGCGCTCACGAACAGTTG |
| Murine *Il-1b* | GAGAATGACCTGTTCTTTGAAGTTG | CTGCCTGAAGCTCTTGTTGA |
| Murine *Il-6* | ACAAAGCCAGAGTCCTTCAG | TGGATGGTCTTGGTCCTTAG |
| Murine *Tnf-α* | CTTCCAGAACTCCAGGCGGT | GGTTTGCTCGACGTGGC |
| Murine *Inf-γ* | GAGGAACTGGCAAAAGGATGG | GTTGCTGATGGCCTGATTGTC |
